# Leveraging Large Language Models to Improve the Readability of German Online Medical Texts: Evaluation Study

**DOI:** 10.2196/77149

**Published:** 2026-01-23

**Authors:** Amela Miftaroski, Richard Zowalla, Martin Wiesner, Monika Pobiruchin

**Affiliations:** 1 Faculty of Informatics, Heilbronn University, Max-Planck-Str. 39, Heilbronn, 74081, Germany, 49 7131 504 ext 633; 2 Research and Innovation Center for Cognitive Service Systems (KODIS), Fraunhofer Institute for Industrial Engineering, Stuttgart, Germany; 3 Consumer Health Informatics Special Interest Group, German Association for Medical Informatics, Biometry and Epidemiology (GMDS) e.V., Cologne, Germany

**Keywords:** artificial intelligence, health information, large language models, patient education material, readability, AI, LLM

## Abstract

**Background:**

Patient education materials (PEMs) found online are often written at a complexity level too high for the average reader, which can hinder understanding and informed decision-making. Large language models (LLMs) may offer a solution by simplifying complex medical texts. To date, little is known about how well LLMs can handle simplification tasks for German-language PEMs.

**Objective:**

The study aims to investigate whether LLMs can increase the readability of German online medical texts to a recommended level.

**Methods:**

A sample of 60 German texts originating from online medical resources was compiled. To improve the readability of these texts, four LLMs were selected and used for text simplification: ChatGPT-3.5, ChatGPT-4o, Microsoft Copilot, and Le Chat. Next, readability scores (Flesch reading ease [FRE] and Wiener Sachtextformel [4th Vienna Formula; WSTF]) of the original texts were computed and compared to the rephrased LLM versions. A Student *t* test for paired samples was used to test the reduction of readability scores, ideally to or lower than the eighth grade level.

**Results:**

Most of the original texts were rated as *difficult* to *quite difficult* (average WSTF 11.24, SD 1.29; FRE 35.92, SD 7.64). On average, the LLMs achieved the following average scores: ChatGPT-3.5 (WSTF 9.96, SD 1.52; FRE 45.04, SD 8.62), ChatGPT-4o (WSTF 10.6, SD 1.37; FRE 39.23, SD 7.45), Microsoft Copilot (WSTF 8.99, SD 1.10; FRE 49.0, SD 6.51), and Le Chat (WSTF 11.71, SD 1.47; FRE 33.72, SD 8.58). ChatGPT-3.5, ChatGPT-40, and Microsoft Copilot showed a statistically significant improvement in readability. However, the *t* tests yielded no statistically significant results for the reduction of scores lower than the eighth grade level.

**Conclusions:**

LLMs can improve the readability of PEMs in German. This moderate improvement can support patients reading PEMs online. LLMs demonstrated their potential to make complex online medical text more accessible to a broader audience by increasing readability. This is the first study to evaluate this for German online medical texts.

## Introduction

### Overview

In the digital era, health information is widely available [1] and exists in many different forms, for example, Wikipedia articles, health-related websites, leaflets, and brochures [2], categorized as patient education materials (PEM). Such materials support medical laypersons in learning about diseases, diagnoses, therapies, etc [3]. Health information should be easy to understand for the general population and to promote health literacy [4]. In this context, the COVID-19 pandemic confirmed the need to improve the general scientific and health literacy [5-7].

However, Zowalla et al [3,8], Rooney et al [9], Yeung et al [5], Gordejeva et al [10], and others have shown that the readability of health information is often unsatisfactory regardless of its source (online material, booklets), authors (official bodies and institutions, individuals), or language. Many of these resources pose a challenge due to their sentence complexity and use of expert language, making it difficult for laypersons to effectively read and understand such material.

Artificial intelligence (AI) offers potential for substantial improvements in health care applications and is becoming omnipresent in recent years [11]. In particular, large language models (LLMs) represent promising opportunities [12,13]. In this context, LLMs can be leveraged to improve the readability of existing PEMs intended for citizens.

Being easily accessible for everyone [14], citizens and patients alike will use them to seek health information online, answers to specific questions, or even therapy advice similar to Internet search engines [15].

For these reasons, citizens will use LLMs to *translate* complex medical terminology and to simplify text material, aiming for an improved comprehensibility [16,17]. Moreover, an increased integration of AI in the curation of health information offers several benefits [18] for institutions, primarily time and cost savings.

### Prior Work

There is a decade-long research tradition about how to use and implement decision support systems, machine learning, and AI solutions in health care. Since 2023, with the widespread availability of LLMs [19], those technologies have been explored for beneficial health care use cases [15] in several medical domains [20-28].

Researchers investigated how publicly available LLMs interfere with patients’ information seeking behavior. Eng et al [29] entered questions about rotator cuff repair surgery in ChatGPT-3.5 and let 2 orthopedic surgeons analyze the answers. The questions were derived from frequently asked questions (FAQs) sites from various patient information websites. The answers by the LLM were evaluated in terms of readability (Flesch-Kincaid grade level); the *Journal of the American Medical Association* Benchmark criteria and the DISCERN score [30] were also used to evaluate reliability and the quality of health information on the internet. The average readability level of the generated answers was equal to a college freshman (Flesch-Kincaid grade of 13.4).

Similar work was conducted by Mika et al [31] who fed ChatGPT with “ten frequently asked questions regarding total hip arthroplasty.” They found that the generated answers were fairly accurate and would be easily understood by patients. Another commonly used readability metric is the Flesch reading ease (FRE) score, which ranges from 0 to 100; lower values indicate a low level of readability, that is, it is difficult to read the text.

Li et al [32] let ChatGPT process 400 English radiology reports (the mean length of words was 164, SD 117). The FRE improved from “38.0±11.8” to “83.5±5.6”.

Similar results were reported by Moons et al [33] who used ChatGPT and Google Bard to reformulate 3 “selected patient information sections from scientific journals.” Google Bard was able to reduce the reading level of the texts to that of sixth graders. However, this was achieved by omission of “significant information” [33].

In an analysis of PEM for bariatric surgery, ChatGPT (version 3.5 and 4) and Google Bard were able to improve the readability levels of 66 frequently asked questions pages of US-based hospitals. The mean FRE scores of the original texts were “48.1 (SD 19.0), which corresponded to ‘difficult to read,’ while initial responses from GPT-3.5, GPT-4.0 and Bard achieved mean scores of 31.4 (SD 11.4), 42.7 (SD 9.7), and 56.3 (SD 11.6), which corresponded to ‘difficult to read,’ ‘difficult to read,’ and ‘fairly difficult to read,’ respectively” [34]. The authors also evaluated the accuracy of the simplified texts. The majority of the LLM responses were equal in accuracy to the original texts, but quality varies among LLMs. Srinivasan et al [34] stress the “importance of evaluating both the readability and quality” of rephrased texts for patient information.

This is also in line with the conclusion by Pal et al [35], who recommend training more specialized LLMs for tasks in the medical domain. They propose that this will add credit and reliability to the answers produced by LLMs in the clinical setting.

Focusing on non-English evaluations, some research was published for expert-centric scenarios: a multilingual benchmark set for answering medical exam questions was developed by the “MedExpQA” study [36]. This contains standardized answers from health experts. To assess the accuracy of medical questions, the study analyzed LLMs with and without retrieval-augmented generation methods. It was found that the models in French, Italian, and Spanish were inferior to those in English. In addition, difficulties such as the tendency to generate incorrect answers and the use of outdated information were identified.

Heilmeyer et al [18] focused on German medical text: they “assessed the feasibility of using nonproprietary LLMs of the GPT variety as writing assistance for medical professionals.” Pretrained LLMs were trained on electronic health records of more than 82,000 patient encounters in a German outpatient clinic. AI tools proved to be “helpful writing assistance” for medical experts and patient reports.

As of today, no readability evaluation has been conducted for LLM-rephrased German health texts from the citizens’ perspective. By *citizens’ perspective*, this study refers to evaluating LLM-rephrased health texts as they would have been experienced by an average layperson without specialized knowledge or expertise in prompt engineering. This approach reflected the realistic scenario of laypersons seeking health information online, using freely accessible tools without systematically optimizing prompts or using application programming interfaces (APIs) to tune LLM model parameters.

### Aims of the Study

The aim of the study is to investigate, from a layperson’s perspective, whether LLMs can simplify and increase the readability of German online medical texts to a recommended level of readability, that is, the eighth grade [37,38].

In this context, 3 specific aims were defined as:

1. Rephrase German medical texts with LLMs,
2. Compute their readability, and
3. Evaluate if the AI-rephrased texts showed a higher level of readability.

## Methods

### Medical Text Corpus

Previous research and a prior sample size calculation (see *Statistical Analysis*) indicated that the desired reduction in Wiener Sachtextformel (4th Vienna Formula) (WSTF) score to meet the recommended grade level could be shown with a rather small sample (n<10). Therefore, a medical text corpus was compiled to represent high prevalence diseases, for example, cancer and diabetes mellitus, and major public health topics, for example, vaccinations, as well as national bodies and institutions such as the national health portals of Germany and Austria or popular online health websites.

For a full list of included content providers and websites, see Multimedia Appendix 1.

First, all 60 websites were visited with a Chrome web browser. Next, the corresponding texts were manually extracted without any HTML-related markup. The 60 plain text of the resulting corpus files were then used for further processing.

### Readability Analysis

The term readability “refers to the properties of written text […] it reflects the (1) complexity of a text’s structure, (2) sentence structure, and (3) chosen vocabulary” [10]. For the German language, 2 well-known readability metrics are established: (1) an adapted version of the (original English) FRE [39] for the German language by Amstad [40] and (2) the WSTF by Bamberger and Vanecek [41]. Both metrics use text parameters like average sentence length and average number of syllables per word.

The FRE score ranges from 0 to 100; lower values indicate a low level of readability, that is, it is difficult to read the text. The WSTF metric ranges from 4 to 15, which roughly corresponds to school grades. For instance, if a text scores a value of 10, at least 10 years in school are necessary for readers to understand this text.

For the readability computations of all texts and to address research aim (2), the analysis framework by Wiesner et al [42] was used. The analysis was conducted on a Windows 10 64-bit computer with Java Runtime Environment (version 18; Oracle Corporation).

### Selection of Large Language Models

A scoping review of well-known code platforms such as GitHub [43] or Hugging Face [44] was conducted to search for available LLMs. In addition, online literature databases such as the Association of Computing Machinery Digital Library and Institute of Electrical and Electronics Engineers Explore were searched to scan publications that already used LLMs (see Table 1).

**Table 1.** Overview of various large language models available as of April 2024.

Name	Year	Domain	Developer	Availability	Open source	Free to use	Language
ChatGPT 3.5 [45]	2022	General	OpenAI	Web	No	Yes	EN^a^
GPT4 [45]	2023	General	OpenAI	Web	No	No	EN
Google Gemini [46]	2023	General	Google	Web	No	Yes	EN
BERT [47]	2018	General	Google	Local	Yes	Yes	EN
Llama 2 [48]	2023	General	Meta	Local	Yes	Yes	EN
Claude 2 [49]	2023	General	Anthropic	Web and Local	No	Yes	EN
T5 [50]	2019	General	Google	Local	Yes	Yes	EN
BLOOM [51]	2022	General	Big Science	Local	Yes	Yes	EN
Microsoft Copilot [52]	2021	General	Microsoft	Web	No	Yes	EN
Falcon LLM [53]	2023	General	Technology Innovation Institute	Local	Yes	Yes	EN
Le Chat [54]	2024	General	Mistral AI	Web	No	Yes	EN
Phönix [55]	2023	General	Matthias Uhlig	Local	Yes	Yes	GER^b^
LeoLM 7b/13b [56]	2023	General	LAION and HessianAI	Web and Local	Yes	Yes	GER
MedAlpaca [57]	2023	Medical	Tianyu Han et al	Local	Yes	Yes	EN
BioMedLM [58]	2024	Biomedical	Stanford CRFM	Local	Yes	Yes	EN

a EN: English.

b GER: German.

Some important aspects and criteria influenced the final selection: The language of the LLM—preferably a German-trained model should be used—as well as the specific field or domain of the LLM (general or medical domain).

Some LLMs can only be executed locally, while some can be used via a web front end. The latter would be preferable because in our use case, LLMs should be used by laypeople, who do not have the hardware capabilities at their homes nor the technical knowledge to host and operate LLMs. Preferably, the use of the LLM should be free of charge and open to use, that is, not in a beta test phase or only available for persons with a technical background (ie, programming skills).

Of 15 candidate LLMs, only 3 met the previously outlined criteria and were selected: (1) ChatGPT 3.5, (2) Microsoft Copilot, and (3) Le Chat. In May 2024 (after the LLM scoping review phase), OpenAI presented and launched their new release: GPT-4o. The authors decided to include this model as well. LeoLM (13b) was initially considered but later excluded due to frequent inaccuracies, very short or context-inadequate outputs, occasional language mismatches (answer in English instead of German), and overall unreliability in handling the text material.

### Accuracy of Rephrased Health Information Texts

The answers generated by each LLM were independently assessed by 3 reviewers (AM, RZ, MP) with a background in medical informatics. Assessments focused on the medical accuracy, clarity, and plausibility of the information provided, ensuring that each response was evaluated not only for linguistic quality but also for its alignment with established medical knowledge and standards. Interrater agreement was measured by calculating Fleiss κ and percent agreement.

### Prompt Engineering

Prompt engineering refers to the process of designing and optimizing the input prompts for any LLM. The structure and content of a prompt can greatly influence the quality of the output generated by the LLM. Today, some techniques have evolved to obtain better results by LLMs:

- *Few-shot prompting* provides examples within a prompt to clarify instructions [59]. This approach improves the model’s ability to interpret and respond accurately to the task, as the examples provided serve to establish patterns and context. The term ‘few’ indicates that a limited number of examples are provided as opposed to zero-shot prompting, where no examples are given.
- *Chain of thought prompting* breaks down complex tasks into steps within a prompt [60]. This approach mimics human problem solving, guiding the LLM through structured reasoning to generate more accurate responses, especially for tasks that require multiple levels of reasoning.
- *Clue and reasoning prompting* introduces a structured reasoning approach [61]. Unlike the other methods, clue and reasoning prompting targets complex linguistic features (eg, irony, contrast, and intensification) and involves 2 stages: the LLM (1) identifies *clues* within the input (eg, keywords, tone, and references) and (2) uses these clues to perform a reasoning process. This step-by-step approach aims to fill gaps in the LLM’s reasoning capabilities, enabling it to deal more effectively with complex linguistic variations.

For the average person seeking health information online, advanced prompting techniques may be too complex to apply. These techniques require understanding how to structure input for LLMs. Few-shot prompting, for instance, involves providing examples within a prompt, requiring users to explain their needs clearly for effective interpretation. Most people would find designing such prompts difficult and time-consuming, especially when simply needing help understanding the provided health information.

For this reason, the authors decided to use a zero-shot prompting approach. Therefore, an extensive role-prompt approach was evaluated with a subset of the medical text corpus (6‐12 texts) and the 3 web-based LLMs. This prompt contained context information and provided a detailed task description for the LLM. However, using this prompt, the results’ readability decreased.

I, a person with no specialist medical knowledge, would like to understand as simply as possible how a stroke is treated by medical staff. I have read a text from gesund.bund.de, which I did not understand because of the medical terminology. Your task as AI ChatBot is to rewrite the following text so that I can understand it completely at the end. Here is the text: {TEXT}

Iteratively, other approaches were tested, eg, English prompt versus German prompt, or prompts with specific instructions to fine-tune a given readability score. Finally, a reduced German role prompt yielded the best results:

A person with no medical knowledge wants to understand a text. You, as a large language model, must simplify the following text for this person using simple language without reducing the content. Here is the text: {TEXT}

Every text and every LLM was input with this prompt, combined with the actual medical text. Due to the limit of 4000 characters for Microsoft Copilot, the texts were split, and several requests were made. Eventually, a total of 240 LLM conversations were conducted between May and July 2024.

### Statistical Analysis

Readability scores for the original and rephrased texts are presented as mean and SD. Student *t* test for paired samples was used to test the reduction of readability scores prior to and after the rephrasing. Prior research of German medical texts [3,10,42] yielded a mean readability of 12.46 (SD 1.84) for the WSTF. This means a reduction of 4.5 grade levels would result in the recommended reading level of 8, that is, a score ≤8.0. Given these parameters, a sample size of 4 would be needed to show such an improvement with an alpha error of 0.05 (adjustment for multiple testing according to the Holm-Bonferroni method [62]) and a power of 95%. Sample size was calculated with G*Power 3.1 [63].

After the analysis of the text corpus’ readability scores, the average readability was calculated as WSTF 11.24 (SD 1.29); FRE 35.92 (SD 7.64). Thus, only a reduction of 3.5 grade levels (for WSTF) would be needed. For the FRE metric, an increase of 25 score points is needed for an eighth grade readability level, that is, a FRE score between 60 and 70.

The hypotheses were formulated as follows:


$$H_{WSTF|0}:\mu_{\text{orig}}-\mu_{\text{LLM}}\leq 3.5$$

$$H_{WSTF|1}:\mu_{\text{orig}}-\mu_{\text{LLM}}§gt;3.5$$

The tests for the FRE metrics were constructed in a similar manner:


$$H_{FRE|0}:\;\mu_{LLM}-\mu_{orig}\leq 25$$

$$H_{\text{FRE}|1}:\mu_{\text{LLM}}-\mu_{\text{orig}}§gt;25$$

In addition, to show if LLMs improved the readability at all, paired *t* tests were conducted. The tests were constructed as follows:


$$H_{WSTF|0}:\;\mu_{orig}\leq \mu_{LLM}$$

$$H_{WSTF|1}:\;\mu_{orig}§gt;\mu_{LLM}$$

For the FRE metrics, the hypotheses were:


$$H_{FRE|0}:\;\mu_{LLM}\leq \mu_{orig}$$

$$H_{FRE|1}:\;\mu_{LLM}§gt;\mu_{orig}$$

## Results

### Readability of the Original Health Information Texts

Most of the original texts were rated as *difficult* to *quite difficult* (average WSTF score 11.24 (SD 1.29), FRE 35.92 (SD 7.64)); this corresponds to 12 years of schooling. The W39 website had the most difficult text (WSTF 13.97, FRE 16.74) to read; the W7 website had the text with the best readability (WSTF 8.70, FRE 51.02). Only 2 websites achieved a grade level of 8 (W7, W9). Table 2 presents the calculated WSTF and FRE scores for the original health information texts with their means and SD.

**Table 2.** Computed readability scores and number of words for 60 medical information texts.

Website	WSTF^a^^,^^b^	FRE^c^^,^^d^	Number of words^e^
W1	9.36	43.93	950
W2	10.63	41.92	1021
W3	10.70	44.26	1007
W4	9.83	41.46	784
W5	10.80	36.40	1909
W6	11.01	41.23	1131
W7	8.70	51.02	907
W8	10.84	34.30	1017
W9	8.90	47.80	1279
W10	10.65	38.82	1434
W11	10.01	43.06	898
W12	12.00	28.65	1214
W13	11.68	31.91	780
W14	10.77	43.18	597
W15	12.28	33.36	1205
W16	9.35	46.21	661
W17	10.32	41.83	780
W18	10.30	44.75	832
W19	10.85	39.14	1321
W20	11.96	29.32	839
W21	11.36	34.43	4225
W22	11.11	34.62	2999
W23	11.93	29.02	114
W24	11.43	34.48	2192
W25	11.55	38.69	1058
W26	9.65	45.50	660
W27	10.93	38.60	425
W28	11.35	29.45	706
W29	11.27	27.70	648
W30	11.85	27.67	562
W31	9.27	46.62	1266
W32	9.17	46.23	2657
W33	10.33	43.09	1306
W34	11.50	35.65	760
W35	9.20	46.04	2672
W36	10.82	36.20	1472
W37	9.57	44.36	1370
W38	11.60	32.86	1173
W39	13.97	16.74	1343
W40	11.90	30.39	1948
W41	11.13	36.13	1678
W42	11.08	37.84	3960
W43	11.35	40.01	794
W44	10.97	37.84	2232
W45	11.87	30.18	1236
W46	13.36	21.97	1527
W47	12.49	27.99	2072
W48	13.66	24.65	2063
W49	12.01	32.93	1117
W50	13.86	22.18	1838
W51	11.62	37.14	762
W52	12.58	31.80	1642
W53	10.22	40.70	516
W54	14.28	19.45	1199
W55	13.88	22.92	1197
W56	12.69	30.66	3383
W57	12.02	32.44	2541
W58	11.41	39.02	746
W59	10.90	36.29	1530
W60	12.03	31.86	2411

a WSTF: Wiener Sachtextformel (4th Vienna Formula).

b WSTF mean 11.24 (SD 1.29).

c FRE: Flesch reading ease.

d FRE mean 35.92 (SD 7.64).

e Number of words, mean 1409 (SD 840).

### Readability of the Rephrased Health Information Texts

Overall, the texts rephrased by the LLMs show an improved readability compared to the original texts. However, the degree of the readability improvements varies greatly.

ChatGPT-3.5 had, on average, a score of 9.96 (SD 1.52) for WSTF, ChatGPT-4o had a mean score of 10.6 (SD 1.37), Microsoft Copilot had a mean score of 8.99 (SD 1.10), and Le Chat had a mean score of 11.7 (SD 1.47). Microsoft Copilot achieved the best readability values compared to the other LLMs (see Table 3).

**Table 3.** Computed readability scores and number of words with mean readability score and SDs, and average differences of original and large language model texts.

	ChatGPT-3.5			ChatGPT-4o			Microsoft Copilot			Le Chat		
Website	WSTF^a^	FRE^b^	Words	WSTF	FRE	Words	WSTF	FRE	Words	WSTF	FRE	Words
W1	9.81	46.17	242	10.25	39.87	496	8.35	51.72	845	10.13	39.35	446
W2	8.38	56.14	286	9.72	43.59	281	8.72	55.38	710	11.27	40.76	798
W3	10.85	41.58	305	11.36	34.35	501	8.60	53.71	817	12.56	34.88	471
W4	9.52	41.80	364	11.78	35.22	370	*7.69^c^*	54.29	610	11.15	36.23	456
W5	9.59	43.00	189	13.17	23.16	273	8.45	49.52	1541	11.84	31.16	914
W6	10.63	45.58	182	12.51	29.02	368	9.27	52.57	841	12.06	36.92	518
W7	8.60	47.11	310	11.97	31.22	565	*6.78*	*60.42*	746	8.60	51.02	540
W8	11.50	31.89	247	10.94	35.65	548	9.51	39.05	839	10.75	33.19	898
W9	*7.01*	55.54	392	11.45	31.89	368	*7.50*	54.58	905	9.09	46.91	884
W10	9.46	45.68	375	11.10	36.70	404	8.10	54.06	1272	12.59	26.46	502
W11	10.97	38.87	246	12.41	32.79	289	*7.80*	57.70	711	13.62	25.75	359
W12	10.01	44.82	278	9.89	44.55	371	10.50	36.75	861	11.08	36.80	385
W13	11.04	39.63	281	9.93	42.85	316	*7.49*	*60.94*	529	12.85	27.51	588
W14	11.58	42.30	195	11.04	42.11	425	8.42	55.94	519	13.13	33.64	433
W15	11.98	38.45	422	11.51	30.80	335	11.16	42.22	1107	13.55	26.82	476
W16	*7.90*	56.36	240	10.24	43.86	403	*7.58*	54.60	518	9.74	47.35	304
W17	10.79	40.32	244	13.18	28.21	414	*7.48*	53.50	462	10.43	41.20	425
W18	*6.86*	*60.56*	328	12.98	29.82	425	8.93	52.24	670	11.42	40.27	694
W19	9.74	46.16	402	11.32	34.63	357	10.18	45.15	987	10.99	39.99	381
W20	10.15	34.17	179	10.23	41.84	371	9.99	41.26	658	11.93	26.95	483
W21	8.64	50.26	569	11.10	34.63	501	11.10	36.22	5170	11.67	33.53	635
W22	10.36	43.12	207	9.90	45.81	485	9.47	44.02	2140	11.67	30.06	358
W23	9.64	43.23	113	10.29	41.88	522	9.31	44.30	171	10.31	36.48	140
W24	13.17	21.91	614	10.28	40.50	527	10.27	40.57	1619	13.51	19.93	603
W25	10.07	50.33	304	9.87	43.50	488	8.97	51.33	633	11.07	41.37	408
W26	*7.98*	56.19	225	*7.86*	50.00	298	*7.68*	57.58	460	10.26	45.04	443
W27	8.76	49.90	336	8.84	41.82	342	*7.04*	58.11	388	9.66	45.03	370
W28	11.32	31.90	268	11.32	31.76	562	9.58	43.31	515	12.69	25.02	350
W29	12.08	27.99	211	11.28	36.53	462	9.35	44.20	456	12.55	22.17	356
W30	11.43	27.47	278	8.09	56.10	467	11.04	34.28	392	11.24	27.05	328
W31	10.45	39.31	305	8.79	46.06	446	8.29	53.77	1067	11.11	36.72	406
W32	*7.94*	55.03	248	9.19	40.50	694	8.13	52.45	1486	9.70	43.85	1543
W33	8.64	50.99	307	10.71	44.53	366	*7.93*	53.66	775	12.79	35.38	344
W34	9.20	48.86	202	10.06	46.79	455	9.45	49.53	743	13.17	36.34	199
W35	8.45	49.27	191	9.99	39.92	472	8.20	51.35	2175	11.12	37.73	1812
W36	*7.45*	58.25	266	*6.69*	59.17	388	8.41	50.82	899	9.77	43.78	351
W37	10.27	46.75	222	9.91	41.15	501	8.04	54.91	1002	11.28	39.32	621
W38	11.02	35.77	207	11.59	36.28	584	10.02	42.72	797	12.54	27.26	343
W39	11.44	38.79	269	*7.86*	54.24	550	9.84	45.85	805	13.95	19.90	331
W40	10.62	35.43	332	9.58	42.17	409	9.71	40.90	1104	14.88	11.25	221
W41	10.51	44.05	266	*7.62*	54.26	356	9.32	48.02	954	11.10	34.85	1022
W42	11.07	44.53	458	9.35	45.18	155	9.04	53.37	2291	11.35	37.16	2792
W43	10.61	43.49	353	*7.74*	55.85	335	8.78	53.93	625	12.40	34.65	378
W44	*7.92*	55.98	222	9.62	39.96	339	8.09	53.08	1405	14.43	16.29	365
W45	8.71	48.01	259	10.31	36.58	391	9.02	47.50	749	9.19	46.47	677
W46	10.94	29.05	214	10.72	35.64	350	11.52	37.48	1260	13.62	20.52	610
W47	9.82	47.68	314	11.44	33.51	436	8.46	52.97	1454	12.09	34.95	428
W48	9.44	42.08	240	10.15	42.42	385	9.34	50.15	1454	13.98	19.24	485
W49	*6.34*	61.81	210	9.54	51.02	450	9.28	45.54	744	11.16	35.22	662
W50	11.44	36.95	180	9.94	42.40	604	10.54	39.41	1294	11.28	36.13	474
W51	10.76	39.57	239	9.73	46.80	442	9.82	45.89	564	11.28	37.34	540
W52	8.66	52.05	253	11.07	39.83	418	10.68	40.98	110	14.48	24.01	387
W53	*6.58*	57.13	166	10.09	42.20	380	*7.69*	55.60	334	9.68	43.02	287
W54	9.87	47.54	224	8.67	51.23	333	9.65	46.25	2380	12.66	29.66	494
W55	*7.92*	52.57	261	9.88	36.97	382	9.84	45.22	867	14.37	18.19	582
W56	11.05	43.55	261	10.36	44.53	414	9.46	46.08	1770	11.45	38.48	797
W57	8.32	50.94	304	10.47	39.10	402	8.74	51.63	899	10.95	31.65	291
W58	*7.89*	56.28	340	10.70	41.63	491	8.01	58.11	566	10.83	40.04	348
W59	8.08	47.49	277	11.75	36.88	539	8.29	48.90	1085	10.81	33.20	733
W60	10.33	44.92	294	9.22	49.62	388	9.76	44.55	1659	11.69	31.57	742
Mean (SD)	9.96 (1.52)	45.04 (8.62)	278 (88)	10.60 (1.37)	39.23 (7.45)	749 (94)	8.99 (1.10)	49.00 (6.51)	1040 (743)	11.71 (1.47)	33.72 (8.58)	570 (406)
DIFF^d^ (DIFF_SD^e^)	1.54 (1.68)	9.13 (8.90)	—^f^	0.93 (2.06)	4.94 (11.78)	—	2.24 (0.98)	13.09 (5.88)	—	−0.47 (1.33)	−2.20 (7.15)	—

a WSTF: Wiener Sachtextformel (4th Vienna Formula).

b FRE: Flesch reading ease.

c Italic font denotes that the target readability (WSTF≤8, FRE≥60) was reached.

d DIFF: difference.

e DIFF_SD: SD difference.

f Not applicable.

Microsoft Copilot achieved the highest average score of 49.0 (SD 6.51) on the readability metric FRE, while Le Chat came last with 33.72 (SD 8.58). ChatGPT-3.5 generated texts with, on average, the fewest words (278, SD 278 words), while Microsoft Copilot generated texts with the most words (1040, SD 743 words) but still less than the original texts.

The ChatGPT-based models (ChatGPT-3.5, ChatGPT-4o, and Microsoft Copilot) achieved an average improvement of 1.54 (SD 1.68), 0.93 (SD 2.06), and 2.24 (SD 0.98) grade levels, respectively, for the WSTF.

**Figure 1.**
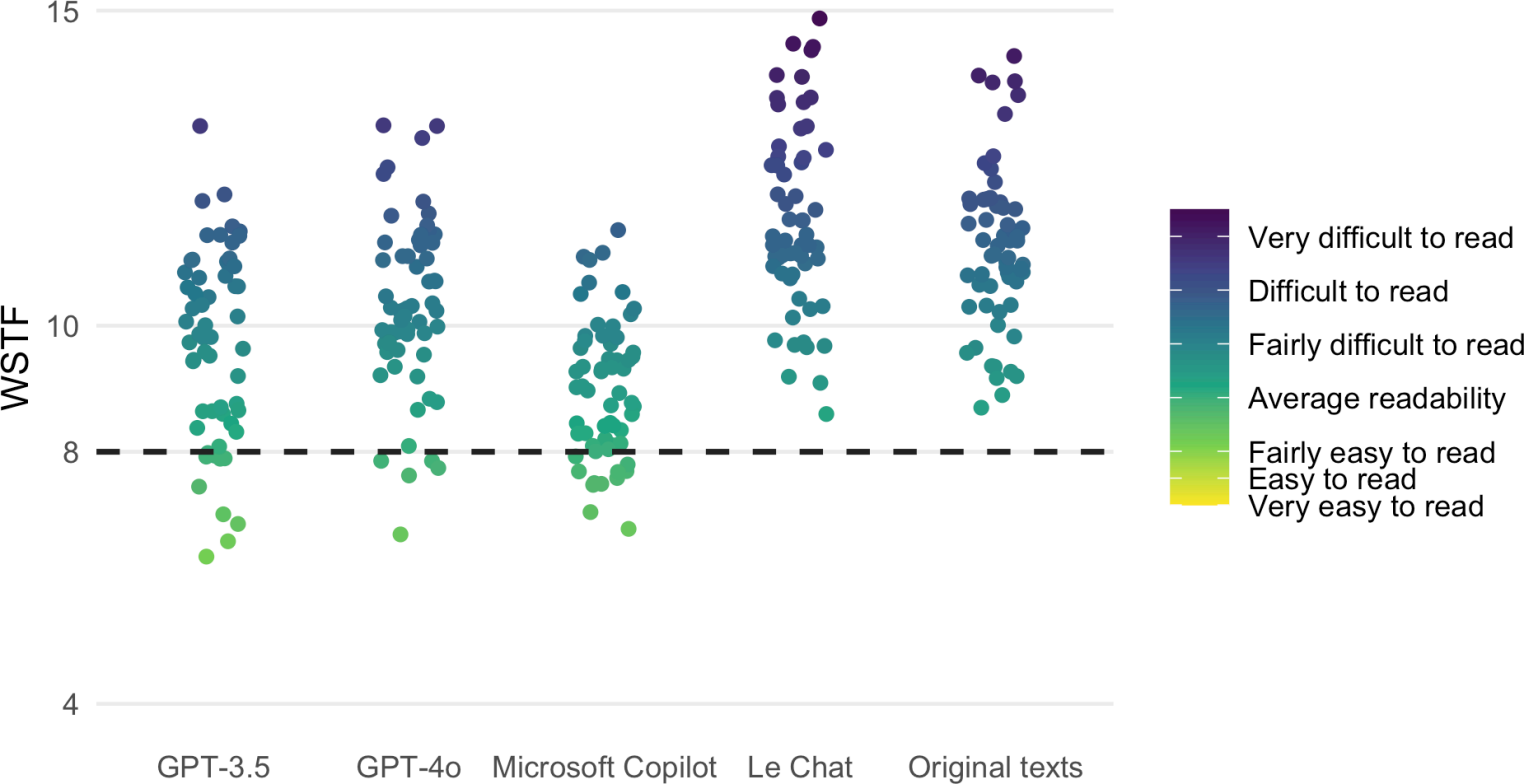
Distribution of calculated WSTF scores for GPT-3.5, GPT-4o, Microsoft Copilot, and Le Chat. The fifth column shows the distribution of the readability scores of the original texts. The dashed line indicates the recommended readability score of 8. WSTF: Wiener Sachtextformel (4th Vienna Formula).

ChatGPT-3.5 reached the desired class level of eighth grade for 20 texts; Microsoft Copilot reached this level for half of the texts (see Table 3 and Figure 1). Notably, the newer ChatGPT-4o achieved this for only 5 texts.

Le Chat did not reach the eighth grade (or lower) for any text. By contrast, the average difference of −0.47 indicates that this LLM tends to decrease the readability. This was also reflected in the statistical tests. For both the WSTF and FRE metrics, the hypotheses that the mean readability improved ($H_{WSTF|1}$ and $H_{FRE|1}$) could not be accepted.

**Figure 2.**
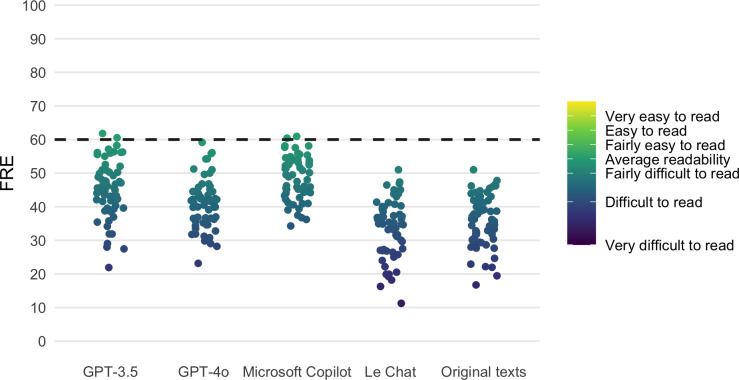
Distribution of calculated FRE scores for GPT-3.5, GPT-4o, Microsoft Copilot, and Le Chat. The fifth column shows the distribution of the readability scores of the original texts. The dashed line indicates the recommended readability score of 60. FRE: Flesch reading ease.

The FRE scores of the rephrased texts improved for GPT-3.5, GPT-4o, and Microsoft Copilot by 9.13, 4.94, and 13.09, respectively (see Table 3 and Figure 2). However, the readability of most of the texts was still low, that is, scores below 60.

On average, Le Chat’s texts were 2.2 scores inferior to the original texts, in line with the evaluation of the WSTF metric.

The findings described above are also reflected in the results of the statistical tests: None of the tests for an improvement to the eighth grade level yielded a significant result, that is, alternative hypotheses could not be accepted. However, except for the Le Chat model, it could be shown that the mean readability was improved significantly, that is, the alternative hypotheses could be accepted. In a nutshell, three out of four LLMs achieved a statistically significant readability improvement, yet it was not high enough to have reached the eighth grade level.

### Accuracy of the Rephrased Health Information Texts

All LLM answers were screened independently by 3 reviewers. Fleiss κ was 0.264, and the percent agreement was 54.6%. This relatively low agreement reflects the difficulty of evaluating medical content without deep domain-level expertise; ideally, assessments would involve medical doctors, and the reliability of the evaluation is further complicated by uncertainty regarding the correctness of the original websites.

Although not a systematic assessment, several obvious mistakes and LLM hallucinations were discovered: Microsoft Copilot shortened the information about endometrial cancer (W29) into “endometrial cancer is the most common cancer among women in Germany” (all the following examples are translated versions of the original German health information texts and rephrased LLM answers). From an epidemiological perspective, this claim is incorrect, with breast cancer being the most prevalent type of cancer among women, constituting a nonnegligible change of meaning in the rephrased text.

The original text about myocarditis (W49) included the sentence: “Myocarditis is also considered to be an important cause of sudden cardiac death in athletes,” which is difficult to understand for readers and may lead to misinterpretations. This kind of sudden cardiac death occurs during exercise, training, or during a match. This information that is not given in the sentence may just be indicated by using the word “athlete.” The rephrased sentence also bears this ambiguity and does even increase it: “When athletes suddenly die, it is often due to inflammation of the heart muscle.” The ‘context’ of sudden death is omitted.

Missing context is also noticed if verbatim speech and statements by medical experts were included in the original texts. The selected LLMs reduced these statements into plain text, thereby omitting the source of the information. For example, the article about myocarditis (W49) included an expert statement as follows: “You should always go to the doctor if you notice symptoms that you are not aware of, says Dr. Milan Dinic, a cardiologist in private practice from Munich.” “Particularly in women, any new complaint between the tip of the nose and the navel is usually heart related. You should therefore definitely think about your heart.”

ChatGPT-3.5 rephrased this to “You should always see a doctor if you notice any new symptoms. In women in particular, many symptoms can indicate heart disease.”

## Discussion

### Principal Results

The original medical texts extracted from health information websites are, on average, *difficult* (for the FRE metric) or *fairly difficult* (for the WSTF) to read. This means that the original texts use complicated sentence structures and/or complex specialist terminology. Our study showed that LLMs can help improve the readability, especially for the models ChatGPT-3.5 and Microsoft Copilot.

ChatGPT-3.5 and Microsoft Copilot were able to reduce text. However, the accuracy of the content must be checked by medical experts to make sure that no ambiguous or false statements were introduced. It is well known that LLMs tend to hallucinate [36,64] or “escalate the minor biases that could occur in the data bank with which it gets trained” [35]. Nevertheless, the authors postulate that the process of “fact-checking” an automatically generated text is more time efficient than manually rewriting medical texts for laypersons. Specialized LLMs or LLMs fine-tuned for medical texts could also be a possible solution to increase the correctness and reliability of generated outputs [35] and thus make this text generation process even more time- and cost-efficient.

The authors found that LLMs moderately increased readability. This is in line with the research by Li [32]. For radiology reports, ChatGPT 3 produces texts that improved the FRE by 45.5 points.

In our analyses, the FRE improvements were 9.13 (ChatGPT-3.5), 4.94 (ChatGPT-4o), and 13.09 (Microsoft Copilot). This might indicate that the rephrasing of texts works better for texts originally written in English. In addition, Srinivasan et al [34] report FRE improvements in a similar range for GPT-3.5 (16.07) and for GPT-4o (5.4).

### Limitations

As the aims of the study were to reflect the experience of an average layperson seeking health information online, no advanced prompt optimization techniques were investigated. While more robust prompts might yield different results, the authors consider it unlikely that nonexpert users would engage in systematic prompt tuning. In addition, reproducibility is hindered by the fact that laypersons won’t experiment with LLM model parameters such as *temperature*. Moreover, tuning model parameters over the chat interfaces isn’t possible in all cases and requires API access. In this context, the authors assume that a high fraction of laypersons do not have the necessary technical background to experiment with LLM APIs and related programming languages.

Additionally, the exact model version of the LLMs used in this study are no longer publicly available. Hence, as in most LLM-based studies, both the selected LLMs and the examined website texts are snapshots in time. The LLM field is evolving rapidly, and reproducibility of the results is difficult.

Another aspect is that the texts taken from the websites may also change over time. The appearance and formatting of the individual web pages were deliberately not considered in this work: Only raw text material was extracted. However, aesthetic and design features or educational multimedia can influence the understandability of information material.

No dedicated *German* LLM was used in this study. It would be interesting to replicate this study with a fine-tuned German LLM. In 2024, the LLM community has a strong focus on English training data and models, and the performance is lower for other languages [36]. Heilmeyer et al [18] noted that specific, on-premise trained German models performed better. However, typical patients or citizens seeking health information will neither have the technical skills or knowledge nor the specialized hardware available to do this on their own.

The systematic evaluation of the (medical) accuracy of rephrased PEMs was beyond the study’s scope, but future interdisciplinary research involving medical experts could address this. Moreover, a follow-up study could more deeply investigate the readability and correctness from a technical point of view by using APIs instead of relying on publicly available chat interfaces. In this context, more recent LLMs could be benchmarked with the same quality-controlled set of text material in an end-to-end evaluation pipeline.

### Comparison With Prior Work

If LLMs were used to answer patient-centric questions about hip arthroplasty, Mika et al [31] report that patients would be able to understand them. However, they do not calculate a readability metric for the given answers and instead rely on a “Response Rating System.” In contrast, Eng et al [29] results confirm the low readability of answers for patient-centric questions.

Compared to the works by [29,31,32,34,65], this study covered a broader spectrum of medical domains: Cancer, cardiovascular conditions, public health topics, etc.

Similar improvements in terms of readability were found by Ovelman et al [66]: They used Claude 2 LLM to create plain language summaries of 10 evidence reviews. The covered topics range from vaccines, prehospital airway management, and malnutrition in hospitalized adults to breast irradiation for breast cancer. For half of their texts, the recommended sixth to eighth grade reading level was met by the generated summaries.

Lyu et al [65] did not measure the quality of the rephrased reports with readability scores but let them be evaluated by experts. In addition, they found that the effect of prompt engineering was not considered high: “All of the five further-modified prompts were found to produce results similar to those of the original prompt and far worse than those of the optimized prompt”.

This study differs from the previously presented evaluations. Here, only German health information texts were rephrased by LLMs and their readability evaluated.

### Innovation

Citizens and patients have been using the Internet for health information seeking for almost two decades. Today, they increasingly consult LLMs in everyday situations: for answers to specific medical questions or for explanations of complex medical texts. This study investigates whether and how LLMs improve the readability of German online medical texts. To the authors’ knowledge, this is the first evaluation of readability metrics for German LLM-rephrased text and original medical text.

Shifting from the perspective of citizens and patients to health professionals or institutions: The use of an LLM could be a time-saving and cost-effective tool to fine-tune their information leaflets, online texts, etc to meet different information needs. The study showed that LLMs are already able to moderately improve readability.

### Conclusions

The use of LLMs can improve the readability of PEMs in the German language but requires careful expert review to ensure accuracy and completeness of medical information. The improvement is rather moderate, averaging 2‐3 school grades (for the WSTF). Still, this improvement can support patients reading PEMs online.

The selection of the LLM seemed critical to achieve good results, whereas a prompt seemed to be less of an influencing factor.

Some rephrased texts conveyed incorrect messages or took statements out of context. This is a serious risk, especially for medical texts. Therefore, a manual check is still needed and advised when using LLMs in similar scenarios.

## Supplementary material

10.2196/77149 Multimedia Appendix 1 List of content providers and websites.

## Abbreviations

AI: artificial intelligence
API: application programming interface
FRE: Flesch reading ease
LLM: large language model
PEM: patient education material
WSTF: Wiener Sachtextformel (4th Vienna Formula)
