# Supplementary material for: Leveraging Large Language Models to Improve the Readability of German Online Medical Texts: Evaluation Study
Source: JMIR AI. 2026 Jan 23;5:e77149. doi: 10.2196/77149 (PMC12829587; doi:10.2196/77149)
Supplement: Multimedia Appendix 1 [file ai-v5-e77149-s001.pdf]

Appendix

A List of content providers and websites

Content providers	Website	Identifier
www.gesundheitsinformation.de	/scharlach.html	W1
	/diabetes-typ-1.html	W2
	/diabetes-typ-2.html	W3
	/schwangerschaftsdiabetes.html	W4
	/brustkrebs.html	W5
	/oertlich-begrenzter-prostatakrebs.html	W6
	/schwarzer-hautkrebs.html	W7
	/gebaermutterhalskrebs.html	W8
	/akne.html	W9
	/kammertachykardie.html	W10
www.gesund.bund.de	/schlaganfall	W11
	/arteriosklerose	W12
	/thromboseneigung-thrombophilie	W13
	/herzinfarkt	W14
	/koronare-herzkrankheit	W15
	/bluthochdruck	W16
	/av-block	W17
	/herzrasen	W18
	/vorhofflimmern	W19
	/akute-herzmuskelentzuendung	W20
www.krebsinformationsdienst.de	/behandlung/operation.php	W21
	/behandlung/strahlentherapie-nuklearmedizin/\linebreak ueberblick.php	W22
	/behandlung/chemotherapie/index.php	W23
	/behandlung/gezielte-krebstherapie.php	W24
	/tumorarten/prostatakrebs/index.php	W25
	/tumorarten/darmkrebs/index.php	W26
	/tumorarten/brustkrebs/index.php	W27
	/tumorarten/gebaermutterhalskrebs/\linebreak index.php	W28
	/tumorarten/endometriumkarzinom/\linebreak index.php	W29
	/tumorarten/hautkrebs.php	W30
www.gesundheit.gv.at	/krankheiten/krebs/brustkrebs/\linebreak frueherkennung.html	W31
	/krankheiten/krebs/hautkrebs.html	W32
	/krankheiten/herz-kreislauf/bluthochdruck/\linebreak hypertonie-was-ist-das.html	W33
	/erste-hilfe/notfall/herzinfarkt.html	W34
	/krankheiten/krebs/hautkrebs.html	W35
	/krankheiten/krebs/darmkrebs.html	W36
	/krankheiten/krebs/brustkrebs.html	W37
	/krankheiten/krebs/info.html	W38
	/krankheiten/krebs/\linebreak bauchspeicheldruesenkrebs1.html	W39
	/krankheiten/krebs/kinder.html	W40
	/krankheiten-symptome/diabetes/typ-1/\linebreak diabetes-mellitus-typ-1-808607.html	W41
	/krankheiten-symptome/diabetes/typ-2/\linebreak diabetes-mellitus-typ-2-808639.html	W42

www.apotheken-umschau.de	/krankheiten-symptome/diabetes/\linebreak symptome-anzeichen-fuer-einen-diabetes-813119.html	W43
	/krankheiten-symptome/herz-kreislauf-erkrankungen/\linebreak niedriger-blutdruck-hypotonie-734171.html	W44
	/krankheiten-symptome/herz-kreislauf-erkrankungen/\linebreak av-block-grade-ursachen-und-therapie-737911.html	W45
	/krankheiten-symptome/herz-kreislauf-erkrankungen/\linebreak herzinnenhautentzuendung-endokarditis-734339.html	W46
	/krankheiten-symptome/herz-kreislauf-erkrankungen/\linebreak herzinfarkt-erkennen-und-behandeln-734303.html	W47
	/krankheiten-symptome/herz-kreislauf-erkrankungen/\linebreak herzrhythmusstoerungen-arrhythmien-735429.html	W48
	/krankheiten-symptome/herz-kreislauf-erkrankungen/\linebreak herzmuskelentzuendung-myokarditis-734381.html	W49
	/krankheiten-symptome/herz-kreislauf-erkrankungen/\linebreak herzbeutelentzuendung-perikarditis-734271.html	W50
www.onmeda.de	/krankheiten/coronavirus/grippe-corona-erkaeltung-id202871/	W51
	/krankheiten/coronavirus/long-covid-id203142/	W52
	/krankheiten/coronavirus/galerie-corona-hausmittel-id212648/	W53
	/krankheiten/herzmuskelentzuendung-id200567/	W54
	/krankheiten/herzrhythmusstoerungen-id201029/	W55
	/krankheiten/herzinfarkt-id200443/	W56
	/krankheiten/herzinsuffizienz-id201000/	W57
	/krankheiten/herzstillstand-id201926/	W58
	/krankheiten/hautkrankheiten/\linebreak hautkrebs-id201608/	W59
	/krankheiten/brustkrebs-id200302/	W60
